# Prospective observational study on the safety of an original fiducial marker insertion for radiotherapy in gynecological cancer by a simple method

**DOI:** 10.1093/jrr/rrz070

**Published:** 2019-10-28

**Authors:** Shuhei Sekii, Kayoko Tsujino, Hikaru Kubota, Satoshi Yamaguchi, Kengo Kosaka, Shuichiro Miyazaki, Nor Shazrina Sulaiman, Yoko Matsumoto, Yosuke Ota, Toshinori Soejima, Ryohei Sasaki

**Affiliations:** 1 Department of Radiation Oncology, Hyogo Cancer Center, Akashi, Hyogo, Japan; 2 Division of Radiation Oncology, Kobe University Graduate School of Medicine, Kobe, Hyogo, Japan; 3 Department of Gynecologic Oncology, Hyogo Cancer Center, Akashi, Hyogo, Japan; 4 Department of Radiology, Hyogo Cancer Center, Akashi, Hyogo, Japan; 5 Department of Radiation Oncology, Hyogo Ion Beam Medical Center Kobe Proton Center, Kobe, Hyogo, Japan

**Keywords:** fiducial marker, titanium, metal, gynecology

## Abstract

Our observational study aimed to verify the safety of our original titanium fiducial markers in gynecological cancer by using a simple insertion method. We prospectively evaluated the safety in patients with gynecological cancer who had undergone our insertion procedure of the titanium markers. The decision to implant a titanium marker was at the discretion of each radiation oncologist. The fiducial markers were manufactured by severing ligating clips for surgery into 3–6 mm pieces and were sterilized thereafter. We inserted an 18-gauge injection needle containing the marker before the marker was extruded by a 22-gauge Cattelan needle or shape memory alloy wire into the tumor or tissues close to the tumor. Severe complications within 3 months after implantation were scored according to the National Cancer Institute’s Common Terminology Criteria for Adverse Events version 4.0. Between August 2016 and December 2018, we enrolled 46 patients. Of 46, 44 underwent implantation. The median age was 58.5 years. The most common primary site was the cervix. Two patients experienced detachment of the markers after implantation. No Grade 3 or higher level of complications was observed. Our simple insertion technique for original titanium fiducial markers was well-tolerated.

## INTRODUCTION

Gynecological carcinoma composed of cervical, uterine, ovarian, vaginal and vulvar carcinoma accounted for approximately 7.4% (1 269 165 out of 17 036 901) of cancers in the world in 2018 [1]. Radiation therapy plays an important role in both curative and palliative treatment of gynecological cancer [2–6].

For the last few decades, application of computed tomography (CT) for planning external beam radiotherapy (EBRT), including 3D conformal radiation therapy and intensity modulated radiation therapy (IMRT) and 3D image-guided brachytherapy (IGBT), have prevailed. A number of situations were encountered when gross tumor volume (GTV) or clinical target volume (CTV) was difficult to determine due to the invisibility of the border with surrounding tissues or the superficial nature of the tumor. To improve these situations, commercially available fiducial markers, such as Acculoc® (Civco, St. Paul, MN, USA), Gold Anchor® (Naslaund Medical AB, Huddinge, Sweden), iGold® (Medikit, Tokyo, Japan) and Visicoil® (IBA dosimetry, Bartlett, TN, USA) have been developed. These products have enabled patient positioning as well as contouring to be performed more precisely and accurately. Also, fiducial markers could be used for monitoring internal motion [7, 8]. However, these products are regulated to be used for specific types of cancer, depending on national insurance criteria, and are not inexpensive because the raw material is gold.

Fiducial markers certificated for use in gynecological cancers are not available in our country (Japan). Hence, we developed alternative fiducial markers by using medical products applicable for other uses. The purpose of our observational study was to verify the safety of our original titanium markers in gynecological cancer using our simple insertion technique.

## MATERIALS AND METHODS

### Study design

Our study was a single institutional prospective observational study. The eligibility criteria were as follows: patients with gynecological cancer including endometrial, vulvar, vaginal and cervical cancer; aged ≥20 years old; Eastern Cooperative Oncology Group (ECOG) Performance Status (PS) ≤ 2; benefit of marker implantation determined at the discretion of each attending radiation oncologist; written informed consent provided by the patient. Patients with coagulation disorder, severe comorbidities, pregnancy or possible pregnancy were excluded from our study. The sample size was not determined by setting alpha and beta errors, but was set up to predict the number of participants enrolled until the scheduled deadline. A total of 75 patients were expected to be accrued in 3 years.

The primary endpoint was the incidence of severe complications within 3 months after the implantation, related to the procedure. Complications were scored according to the National Cancer Institute’s Common Terminology Criteria for Adverse Events (CTCAE) version 4.0. Grade 3 or higher complications were regarded as severe. The threshold of the endpoint was 5% of eligible patients. When <5% experienced grade 3 or higher complications, our procedure was considered to be safe.

### Procedural method

**Fig. 1:**
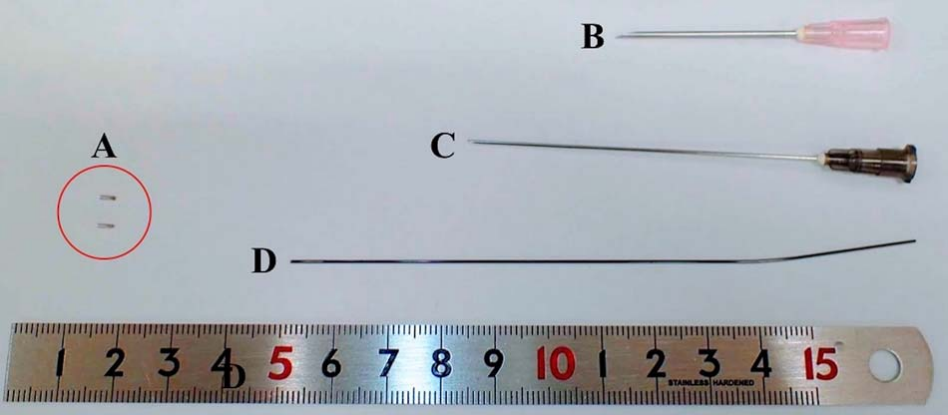
The main apparatus used for the implantation: (**A**) our original titanium fiducial markers, (**B**) 18-gauge injection needle, (**C**) 22-gauge Cattelan needle, and (**D**) shape memory alloy wire.

**Fig. 2:**
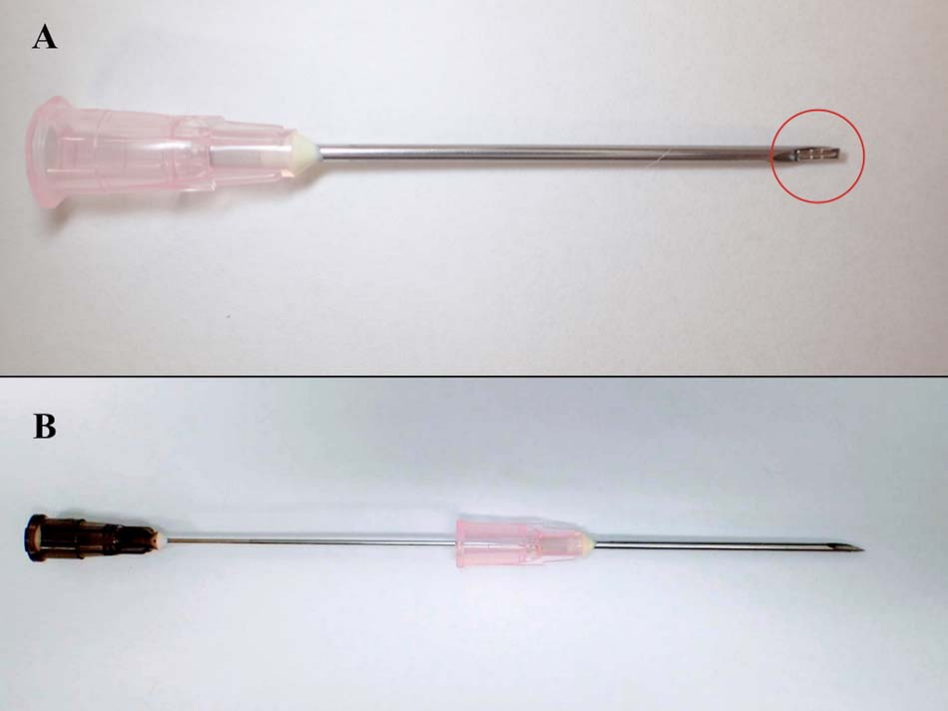
Method of intruding and extruding the fiducial marker. (**A**) Marker was placed on the bevel of an 18-gauge injection needle by forceps to intrude onto the shaft. (**B**) After insertion of the needle into the tumor or tissue close to the tumor, the marker was extruded into by a 22-gauge longer needle or shape memory alloy wire from the hub of the 18-gauge injection needle.

**Fig. 3:**
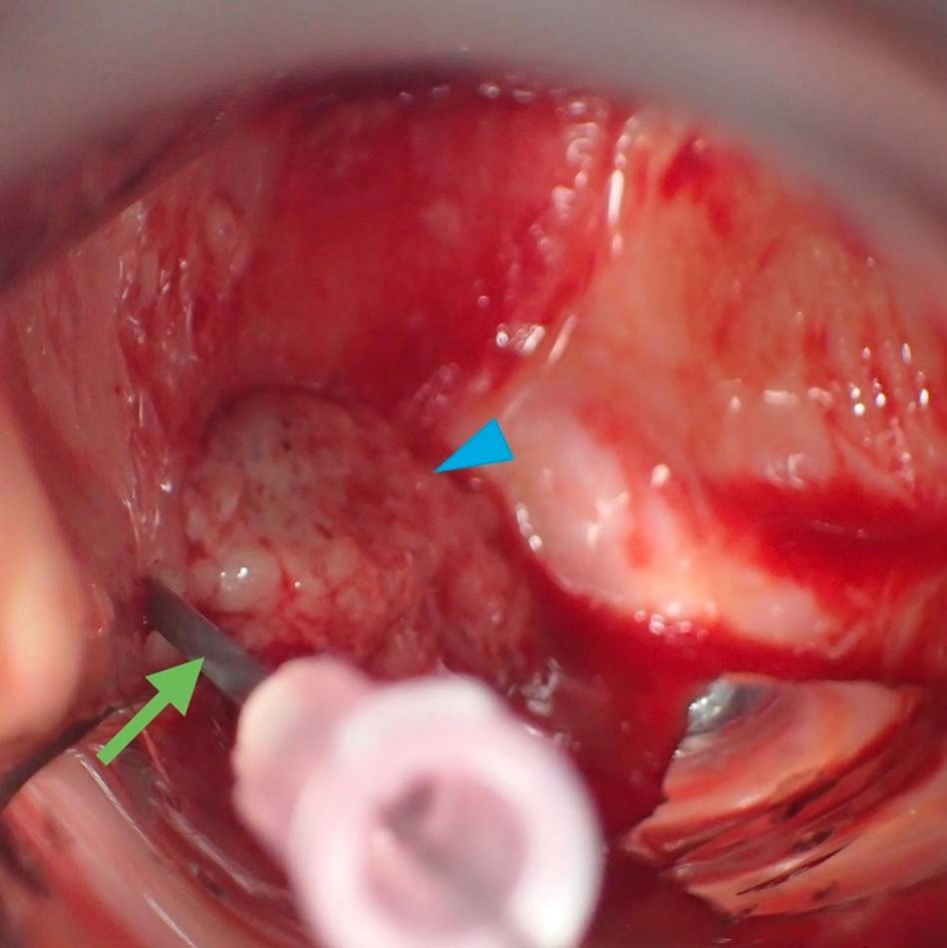
Insertion of marker in a patient with vaginal cancer. Insertion of an 18-gauge injection needle (arrow) at the right caudal side of the vaginal tumor (arrow head).

Medium-sized ligation clips (Weck Ligation Clip®, Teleflex Inc., Wyne, PA, USA) made of titanium, which were originally used for hemostasis during surgery, were selected as materials for the development of the marker. Our markers were manufactured by severing these clips into 3–6 mm pieces (Fig. 1A) and were sterilized thereafter. When implanting, we placed the marker on the bevel of an 18-gauge injection needle (Fig. 1B) to insert the marker into the shaft of the needle (Fig. 2A) before the marker was extruded into the tumor or the tissue close to the tumor by a 22-gauge longer needle (Fig. 1C) or shape memory alloy wire (Fig. 1D) from the hub of the 18-gauge needle (Fig. 2B). Figure 3 shows the case of a patient with vaginal cancer who underwent the marker implantation at the right caudal side of the vaginal tumor.

### Imaging and clinical follow-up

**Fig. 4:**
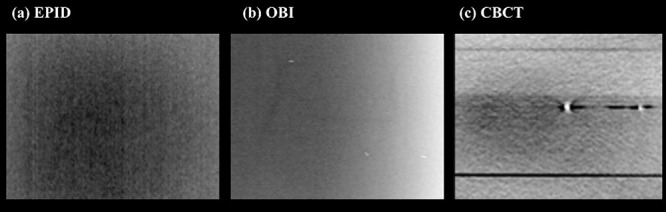
Images of our original titanium fiducial marker obtained by (**a**) electronic portal imaging device (EPID), (**b**) on-board imager (OBI), and (**c**) cone-beam CT (CBCT). The marker could be detected by OBI and CBCT, although it was not detected by EPID.

Before initial application, one medical physicist (K.K.) evaluated the visibility of the titanium marker. The marker was interposed between solid phantoms with 20 cm thickness evaluated by an on-board imager (OBI, Varian Medical Systems, Palo Alto, CA, USA), cone-beam CT (CBCT) and an electronic portal imaging device (EPID). The marker could not be detected by EPID, but was identified by OBI and CBCT (Fig. 4). Given the results, the markers could be determined to be clinically applied, because we usually used CBCT for patient positioning when delivering EBRT of gynecological carcinoma.

Magnetic resonance imaging (MRI) (Signa HDxt, GE Medical Systems, Tokyo, Japan, and Ingenia 3.0 T, Philips Healthcare, Best, The Netherlands) was done in the patients who would receive brachytherapy after implantation unless contraindicated. Patients were closely followed up every 1–3 months for the initial 2 years, every 3–6 months after 2 years. During follow up, MRI was performed at the discretion of physicians.

### Ethical consideration

Our study was approved by the institutional ethical review board and registered with UMIN-CTR, Identifier 000023449.

## RESULTS

Between August 2016 and December 2018, 46 patients participated in the study. Two patients out of 46 did not undergo implantation: one patient had metal allergy and the other patient did not require implantation after the consent. Therefore, we analyzed 44 patients. Patient accrual was terminated earlier than planned because we considered it difficult to continue the study due to a new strict law against clinical trials involving human beings. The median (interquartile range, IQR) follow-up period was 14.0 months (10.8–20.3). The median (IQR) age was 58.5 years (43.0–68.0). An average of 1.2 markers were used. Table 1 summarizes the clinical characteristics of the patients. More than three-quarters of the patients had cervical cancer. The most common reasons for the implantation were to help delineation of GTV or CTV in either EBRT or BT planning, followed by monitoring internal motion. Therefore, the most common site implanted was the vaginal vault. In one case, four markers were implanted into the tumor to be used as a reference for daily correction of applicator deviation. This was because the patient received interstitial BT twice daily at 6-h intervals with patients kept in bed overnight throughout the treatment period. The median time interval (IQR) between implantation and the last day of whole radiotherapy was 57 days (54–62). In two cases, the detachment of markers was confirmed *in situ* on CBCT. In one case, the marker dropped out after 2 weeks possibly because it was not properly implanted, and in another case the marker dropped out 3 weeks after the implantation possibly because of tumor shrinkage.

**Table 1:** Clinical characteristics

Age (years)		
	Median	58.5
	IQR	43–68
ECOG PS (n)		
	0	35
	1	8
	2	1
Primary site (n)		
	Cervix	38
	Corpus	2
	Vagina	4
Treatment setting (n)	Definitive (primary/recurrence)	21/4
	Postoperative	19
Reason for implantation (overlapping included) (n)	Aid to delineate GTV or CTV	43
	Assess intra- or inter-fractional motion	20
	Reference for daily correction during ISBT	1
Time interval between implantation of fiducial marker and the last day of radiotherapy (days)	Median	57
	IQR	54–62
Number of fiducial markers implanted for each patient (n)	1	36
	2	7
	4	1
Number of fiducial markers in each implanted site (n)	In vaginal vault	20
	Around vaginal disease^*^	29
	In tumor	5

^*^Vaginal disease included vaginal invasion of cervical cancer or vaginal cancer.

IQR = interquartile range, PS = performance status, GTV = gross tumor volume, CTV = clinical target volume, ISBT = interstitial brachytherapy.

None of the patients experienced Grade 3 or higher complications within 3 months after the implantation. Grade 2 hematoma on the vaginal vault was observed in one patient 2 weeks after implantation into the vaginal vault (Table 2). Because the excised hematoma was histologically proved to be squamous-cell carcinoma and the marker was not located in the hematoma, the relationship between the procedure and the hematoma formation was determined as reasonable. Therefore, our primary endpoint was met. Also, there were no complications thereafter until the last follow-up in the study cohort. Half of the patients underwent 1.5- or 3-Tesla MRI after the implantation for a cumulative total of 54 times. No adverse effects related to MRI were observed.

## DISCUSSION

In our study, our original fiducial markers inserted using the simple insertion method were found to be well-tolerated. No severe procedure-related complications were observed, suggesting that these markers using our method may be applicable to daily clinical practice. In one case, hematoma was observed after implantation. We considered that the hematoma formation was caused by a recurrent tumor because the patient had no coagulation disorder and the pathological diagnosis showed that the hematoma was proved to be squamous cell carcinoma. In one case out of two cases of marker detachment, the marker was assumed to be unstable because the tumor where the marker was inserted was necrotic, and the detachment occurred as the tumor shrank. Therefore, although our study included a limited number of cases where the marker was implanted into a tumor, we considered that it was possible to implant into a tumor for a long period by avoiding necrotic areas of the tumor.

Fiducial markers are helpful for detecting the area of superficial tumor and for patient positioning. Within the field of radiotherapy for gynecological cancer, various materials have been used as fiducial markers. Kaatee *et al.* assessed internal cervix movement by using tantalum markers [9]. TraceIT® (Augmenix, Bedford, MA, USA) is a product, recently approved by the Food and Drug Administration in the USA, that includes iodinated polyethylene glycol hydrogel. The first report on this product demonstrated its visibility and ease of handling without acute complications [10].

Our original markers are useful for brachytherapy of gynecologic cancer. In one case we used the original markers as useful references for daily correction in a patient who underwent multi-catheter interstitial brachytherapy; it was necessary to keep patients in bed overnight throughout the treatment period. Regarding vaginal recurrence of cervical cancer or endometrial cancer of which brachytherapy with EBRT is an effective treatment [11–14], the markers are also helpful for contouring GTV in brachytherapy as with EBRT planning, considering tumor shrinkage after EBRT.

Gold has been selected as the main raw material in several commercially available fiducial markers because of its stability and X-ray visibility; however, the unit cost of gold was reflected in the price of these fiducial markers. Moreover, the use of gold markers is restricted to a particular type of carcinoma depending on the country or institution. Our original inexpensive markers, the materials for which are easily available, solved these issues, especially in the field of gynecological cancer. The markers had the advantage of being compatible with MRI, given the non-ferromagnetic property of titanium, whereas they had the disadvantage of being difficult to identify due to their small size and poor signal on MRI. Therefore, although a few national surveys have demonstrated the increasing application of 3D IGBT in radiotherapy for patients with cervical cancer [15–17], our markers were useful only when planning was done based on the fusion images generated using MRI and CT in MRI IGBT.

**Table 2:** Complication details after implantation of the original markers

	*n*	Detail
Grade 2 hematoma	1	Hematoma was observed 15 days after implantation in a 53-year-old patient, who had undergone hysterectomy and was scheduled to receive postoperative radiotherapy. Pathological examination of excised hematoma revealed squamous cell carcinoma.

A limitation of the study was that it was conducted at a single institution. The patient enrollment was terminated earlier than planned because of new strict laws against clinical trials, thus the total number of patients included in the study was smaller than initially planned. However, because this study was only an observational study, the initially planned sample size was not calculated based on alpha and beta errors. Given there were no Grade 3 toxicities related to fiducial marker insertion, which defined as an event, it can be said that important findings were derived from this study despite the reduction in the sample size of the study. Therefore, despite the reduction in the sample size of this study, it can be said that important findings were derived from it. Our original marker using the simple implantation method would be useful in the field of radiotherapy for gynecological cancer, especially in institutions with scarce medical resources. In conclusion, our original titanium markers implanted using the insertion technique proposed herein were well-tolerated.
